# Sex Differences in the Impact of Obstructive Sleep Apnea on Glucose Metabolism

**DOI:** 10.3389/fendo.2018.00376

**Published:** 2018-07-10

**Authors:** Karla A. Temple, Rachel Leproult, Lisa Morselli, David A. Ehrmann, Eve Van Cauter, Babak Mokhlesi

**Affiliations:** ^1^Department of Medicine, Sleep, Metabolism and Health Center, University of Chicago, Chicago, IL, United States; ^2^Section of Endocrinology, Diabetes and Metabolism, Department of Medicine, University of Chicago, Chicago, IL, United States; ^3^Division of Endocrinology and Metabolism, Department of Internal Medicine, University of Iowa Carver College of Medicine, Iowa City, IA, United States; ^4^Section of Pulmonary and Critical Care, Department of Medicine, Sleep Disorders Center, University of Chicago, Chicago, IL, United States

**Keywords:** obstructive sleep apnea, sleep-disordered breathing, diabetes, glucose tolerance, insulin resistance, beta-cell, gender, sex

## Abstract

**Objectives:** Obstructive sleep apnea (OSA) is more prevalent in men and is an independent risk factor for type 2 diabetes. We aimed to determine if there are sex differences in the impact of OSA on glucose metabolism in nondiabetic overweight and obese adults.

**Methods:** One hundred and forty-five men and women (age 33.4 ± 0.6, BMI 37.2 ± 0.7, 70.3% blacks) from the community underwent in-laboratory polysomnography. Severity of OSA was assessed by the apnea-hypopnea index (AHI). Glucose tolerance was assessed using fasting glucose, 1-h glucose, 2-h glucose and the area under the curve (AUC) during the 2-h oral glucose tolerance test (OGTT). Fasting insulin resistance was assessed by HOMA-IR, and insulin sensitivity during the OGTT was assessed by the Matsuda Index. Pancreatic beta-cell function was assessed by fasting HOMA-%B and by AUC_insulin/glucose_, insulinogenic index, and oral disposition index (DI_oral_) during the OGTT. All comparisons were adjusted for age, BMI, race and severity of OSA.

**Results:** There were no significant demographic differences between men and women without OSA. Men and women with OSA were similar in age, BMI, and severity of OSA, but there were more black women with OSA. Compared to women with OSA, men with OSA had significantly higher fasting glucose, 1-h glucose levels, AUC_glucose_, and AUC for insulin secretion rate (AUC_ISR_) but similar 2-h glucose levels. These differences persisted in adjusted analyses. Men with OSA secreted significantly more insulin than women with OSA in order to achieve similar glucose levels. Men with OSA had significantly worse beta cell function as measured by the DI_oral_ than women with OSA. In contrast, there were no significant sex differences in measures of glucose tolerance and beta-cell function in participants without OSA.

**Conclusion:** Men with OSA secreted more insulin compared to women with OSA in order to maintain glucose homeostasis. The adverse impact of OSA on beta-cell responsiveness was larger in men, which may result in an overall greater risk of type 2 diabetes compared to women.

## Introduction

Type 2 diabetes affects nearly 30 million individuals or 9.4% of the US population with an estimated 1.5 million new cases per year. Even more alarming is the significant increase in the prevalence of prediabetes (impaired fasting glucose and/or impaired glucose tolerance), an intermediate state between normal glucose tolerance and overt diabetes. According to the Centers for Disease Control and Prevention, the number of American adults with prediabetes increased from 57 million in 2008 to 84 million in 2015 (1). Undoubtedly the high prevalence of obesity has played a pivotal role in this epidemic. In parallel, the obesity epidemic has also resulted in an increased prevalence of obstructive sleep apnea (OSA) in the general population (2, 3).

Several clinic-based and community-based cross-sectional studies have found a robust association between the presence and severity of OSA—as measured by the apnea-hypopnea index or AHI—and insulin resistance in both men and women, independent of age and various measures of adiposity (4–10). A recent meta-analysis of 9 longitudinal studies that included 64,101 participants, with follow up ranging from 2.7 to 12.8 years, revealed that OSA is associated with incident type 2 diabetes with an adjusted pooled relative risk of 1.35 (95% CI, 1.24–1.47) (11). However, the sex-specific difference of the impact of OSA on glucose metabolism remains mostly unexplored. To that end, we aimed to quantify the impact of OSA on glucose tolerance, insulin sensitivity, beta-cell responsiveness and diabetes risk in a community-based cohort of overweight and obese men and women without diabetes.

## Methods

### Participants

Subjects were recruited using flyers and public advertisement. The flyer requested healthy obese men and women between ages 18–50 to volunteer for a research study related to sleep and metabolism. Interested participants who called in to the recruitment phone line underwent a brief phone screen to assess eligibility for the study. Eligible participants were admitted to the University of Chicago General Clinical Resource Center. All subjects were between 18 and 50 years of age, with a body mass index (BMI) >25 kg/m^2^, and free of psychiatric, endocrine and cardiovascular disorders except for well-controlled hypothyroidism and hypertension. Sleep complaints or symptoms of OSA were not used as selection criteria for the study. Shift workers, subjects with chronic insomnia, and subjects with self-reported habitual sleep duration of <6.5 h per night or more than 9 h per night as well as any subjects with diagnosis of a sleep disorder other than OSA were excluded. Other exclusion criteria included any prior or current treatment for OSA (upper airway surgery, CPAP therapy, oral appliances or supplemental oxygen), active cigarette smoking, habitual alcohol intake above 2 drinks per day, previous diagnosis of type 2 diabetes, use of antihypertensives that impact sleep or glucose metabolism (e.g., thiazide diuretics and beta blockers), caffeine intake above 300 mg per day, pregnancy, and women taking hormonal therapy. We also excluded post-menopausal women and women with established diagnosis of polycystic ovary syndrome (PCOS) or suspicion of PCOS based on hyperandrogenemia. All study participants gave written informed consent prior to participating in this study. This study was approved by the University of Chicago Institutional Review Board, and was conducted in accordance with the Declaration of Helsinki.

Consented subjects had a physical examination, and a complete medical history was obtained. Height and weight were measured in all participants on the night of the polysomnography (PSG). Race was self-reported and categorized as non-Hispanic white or black. Subjects had an overnight in-laboratory PSG to assess the presence and severity of OSA. The following morning, a standard 75-g oral glucose tolerance test (OGTT) was performed to measure glucose tolerance and insulin sensitivity.

### Polysomnography

Subjects were admitted to the University of Chicago Clinical Resource Center (CRC) and underwent an overnight in-laboratory PSG. Lights were turned off at 11 pm and turned on at 7 am. The PSG (Neurofax EEG 1100 system; Nihon Kohden, Foothill Ranch, CA) included recordings of six electroencephalogram channels, bilateral electro-oculograms, chin and tibialis electromyogram, electrocardiogram, airflow by nasal pressure transducer and oronasal thermocouples, chest and abdominal wall motion by piezo electrodes, and oxygen saturation by pulse oximeter. All PSGs were staged and scored according to the 2007 American Academy of Sleep Medicine Manual for the Scoring of Sleep and Related Events (12). Apneas were defined as total cessation of airflow for at least 10 s (obstructive if respiratory effort was present and central if respiratory effort was absent). Hypopneas were scored if the magnitude of ventilation signal decreased by at least 50% of the baseline amplitude of the nasal pressure transducer for at least 10 s and were associated with either a 3% or greater drop in oxygen saturation as measured by finger pulse oximetry, or an electroencephalographic microarousal (12). AHI was defined as the total number of obstructive apneas and obstructive hypopneas per hour of sleep. Severity of OSA was measured by the AHI. A subject was considered not to have OSA if the AHI was <5, to have mild OSA if the AHI was 5–14, moderate OSA if the AHI was 15–29, and severe OSA if the AHI was ≥30. The oxygen desaturation index (ODI) was defined as the total number of oxygen desaturations of at least 3% per total sleep time (TST) in hours. The microarousal index (MAI) was calculated as the total number of microarousals per hour of sleep.

### Oral glucose tolerance test (OGTT)

After a 12-h overnight fast, an intravenous catheter was placed into an antecubital vein for blood drawing. Baseline blood samples were drawn at −15 and 0 min for measurement of glucose, insulin, and C-peptide concentrations. At time 0 min, subjects consumed a 75-g glucose beverage over a period not to exceed 5 min. Subsequent blood samples were drawn at 30, 60, 90, and 120 min for measurement of glucose, insulin, and C-peptide concentrations. If the fasting glucose concentration was ≥100 mg/dl but <126 mg/dl, a diagnosis of impaired fasting glucose (IFG) was assigned; a fasting glucose concentration >126 mg/dl was diagnostic of type 2 diabetes. The glucose concentration post-2h glucose challenge was used to diagnose normal glucose tolerance (<140 mg/dL), impaired glucose tolerance (IGT; 140–199 mg/dl) and type 2 diabetes ≥200 mg/dL) (13). Area under the curve (AUC) for glucose and insulin response was calculated for the first 2-h interval after glucose load using the trapezoidal rule (14, 15).

The degree of insulin resistance was quantified using the homeostasis model assessment index of insulin resistance (HOMA-IR) [(glucose (mmol/L) • insulin (mIU/L))/22.5]. (16) Fasting HOMA-IR and area under the curve of glucose (AUC glucose) were used as measures of insulin resistance and glucose tolerance, respectively. The Matsuda Index was used as a measure of insulin sensitivity (17). Beta-cell responsiveness was assessed using the fasting HOMA-%B [(20 • insulin (mIU/L))/(glucose (mmol/L) – 3.5)], AUC_insulin_/AUC_glucose_ [AUC_insulin/glucose_] (16), AUC_ISR_, and insulinogenic index (IGI) (18). Oral disposition index (DI_Oral_) was used as a measure of beta-cell function adjusted for insulin sensitivity (18).

### Assays

Plasma glucose was assayed by the glucose oxidase method (YSI Life Sciences). Serum insulin and C-Peptide were measured by chemiluminescence assays using the Immulite immunochemistry system (Diagnostic Products Corp., Los Angeles, CA). As a result of hemolysis, <1.5% of insulin values were adjusted by linear interpolation or extrapolation after examination of corresponding C-Peptide values. Hemoglobin A1c was measured using turbidimetric inhibition immunoassay (Roche Diagnostics).

### Determination of insulin secretory rates

In each blood sampling interval during the OGTT, the insulin secretion rate (ISR) (19) was mathematically derived from plasma C-Peptide levels using a two-compartment model for C-Peptide disappearance kinetics (20, 21). The kinetic parameters were obtained from published demographic data taking into account sex, age, and body surface area (22). The mean (±SEM) parameter values were 4.55 ± 0.0 min for the short half-life, 33.8 ± 0.08 min for the long half-life, and 0.78 ± 0.0 for the fraction of decay associated with the short half-life. The volume of distribution averaged 4.69 ± 0.05 L.

### Statistical analyses

Continuous variables are expressed as mean ± SEM for normally distributed data, or median with 25–75% interquartile range (IQR) when the assumption of normality was not met; categorical variables are summarized as percentages (%). The main objective of this study was to determine if sex differences exist in the impact of OSA on insulin resistance and glucose tolerance (fasting HOMA-IR, AUC glucose, and Matsuda Index) and beta-cell function (HOMA-%B, AUC_insulin/glucose_, insulinogenic index and oral disposition index). We therefore created four groups based on sex (men, women) and OSA (presence, absence). Comparisons were made separately between men and women with OSA and men and women without OSA. Unadjusted group comparisons were performed using a *t*-test for normally distributed continuous variables and the Wilcoxon/Mann-Whitney test for non-normally distributed continuous variables. Pearson Chi Square test was used to compare categorical variables. Multivariate linear regression models were constructed to examine whether sex was independently associated with measures of glucose metabolism after adjusting for age and BMI as continuous variables, race, and severity of OSA based on the natural log of AHI (*Ln*AHI). Dependent variables that were not normally distributed were log transformed. Given that the calculation for insulin secretion rate (ISR) takes into account body surface area, the multivariate regression models examining AUC_ISR_ did not adjust for BMI. For AHI values equal to zero, we used the formula Ln (AHI + 0.1) to log transform. *P*-values <0.05 were considered statistically significant. All statistical calculations were performed using JMP 9.0 statistical software for Macintosh (SAS Institute).

## Results

A total of 157 subjects participated in the study. An OGTT was not performed in 6 subjects who had a fasting plasma glucose ≥126 mg/dl, consistent with the presence of undiagnosed type 2 diabetes. Four additional subjects were excluded because their HbA1c level was ≥6.5%. Two subjects were excluded because of uncontrolled hypothyroidism. Thus the final analytic cohort included 145 subjects (90 women and 55 men). OSA, defined as AHI ≥5 events per hour, was present in 72.7% of men and 43.3% of women (*p* < 0.001). We categorized the subjects by sex and presence of OSA. Table 1 summarizes the demographics of the four groups of subjects. There were no significant differences between men and women without OSA. In those with OSA, men and women had similar age and BMI, but there were more black women than black men with OSA (*p* = 0.0016).

**Table 1:** Subject demographics.

**Variable**	**Women without OSA** **(*n* = 51)**	**Men without OSA** **(*n* = 15)**	p^*^ value **(*n* = 66)**	**Women with OSA** **(*n* = 39)**	**Men with OSA** **(*n* = 40)**	p^*^ value **(*n* = 79)**
Age (years)	30.2 ± 0.7	27.6 ± 1.6	0.091	36.0 ± 1.3	37.1 ± 1.0	0.485
BMI (kg/m^2^)	35.6 ± 0.9	35.0 ± 1.6	0.757	40.3 ± 1.5	37.3 ± 1.4	0.152
Blacks, *n* (%)	37 (72.5)	9 (60.0)	0.353	34 (87.2)	22 (55.0)	**0.0016**
Non-hispanic White, *n* (%)	14 (27.5)	6 (40.0)		5 (12.8)	18 (45.0)	

Data are given as mean ± SEM or n (%)

* *p-values reflect student's t-test for continuous variables and Pearson coefficient for categorical variables. P-values in bold are statistically significant*.

Table 2 summarizes polysomnographic differences between men and women with and without OSA. There was a statistically significant, but likely clinically less relevant, difference in AHI between men and women without OSA (1.9 vs. 1.1; *p* = 0.0265). In participants with OSA, the only polysomnographic difference between men and women was a lower percentage of slow wave sleep in men (*p* = 0.0017). Importantly, the severity of OSA was not significantly different between men and women with OSA.

**Table 2:** Polysomnographic variables.

**Variable**	**Women without OSA** **(*n* = 51)**	**Men without OSA** **(*n* = 15)**	*p-*value^*^ **(*n* = 66)**	**Women with OSA** **(*n* = 39)**	**Men with OSA** **(*n* = 40)**	*p-*value^*^ **(*n* = 79)**
Total sleep time (minutes)	447 (422–460)	454 (433-491)	0.239	434 (393-450)	422 (377-457)	0.791
Sleep efficiency (%)	91.4 (85.9–94.5)	94.3 (85.7–96.4)	0.449	85.6 (82.7–92.4)	86.7 (81.0–92.7)	0.961
Slow wave sleep (%)	11.6 (5.7-16.3)	5.6 (2.0–13.9)	0.076	9.1 (3.6–14.2)	1.9 (0.1–9.9)	**0.0017**
REM sleep (%)	23.5 (20.6–26.2)	22.5 (17.8–26.7)	0.245	19.9 (16.6–23.5)	22.2 (17.8–26.3)	0.109
AHI	1.1 (0.5–2.3)	1.9 (1.7–2.6)	**0.0265**	15.2 (7.3–21.7)	18.4 (11.8–28.5)	0.096
REM AHI	1.9 (0.6–4.1)	2.8 (1.3–5.3)	0.229	22.3 (5.8–37.2)	23.8 (14.2–51.4)	0.281
NREM AHI	0.7 (0.2–2.2)	1.4 (0.8–3.0)	**0.0187**	12.8 (7.4–21.6)	16.6 (8.6–25.1)	0.184
MAI	10.6 (7.4–14.5)	10.7 (7.3–14.1)	0.748	19.2 (17.0–26.7)	23.5 (13.4–29.3)	0.764

Data are given as median (interquartile range).

* *p-values are from Wilcoxon test. P-values in bold are statistically significant*. *REM, Rapid Eye Movement; AHI, Apnea-Hypopnea Index; NREM, non-Rapid Eye Movement; MAI, Microarousal Index*.

The proportion of individuals with IFG varied between the 4 groups (*p* = 0.0004). A higher prevalence of IFG was observed in men with and without OSA (35.0 and 26.7%, respectively) compared to women with and without OSA (10.3 and 3.9%, respectively). After adjusting for age, BMI, race, and severity of OSA (*Ln*AHI), men were more likely to have IFG (odds ratio 10.2, CI 2.9–42.7; *p* = 0.0001). The prevalence of impaired glucose tolerance (IGT) was higher in men and women with OSA (27.5 and 20.5%, respectively) compared to men and women without OSA (6.7 and 9.8%, respectively). After adjusting for confounders, there was no significant group differences for IGT (*p* = 0.094).

**Figure 1:**
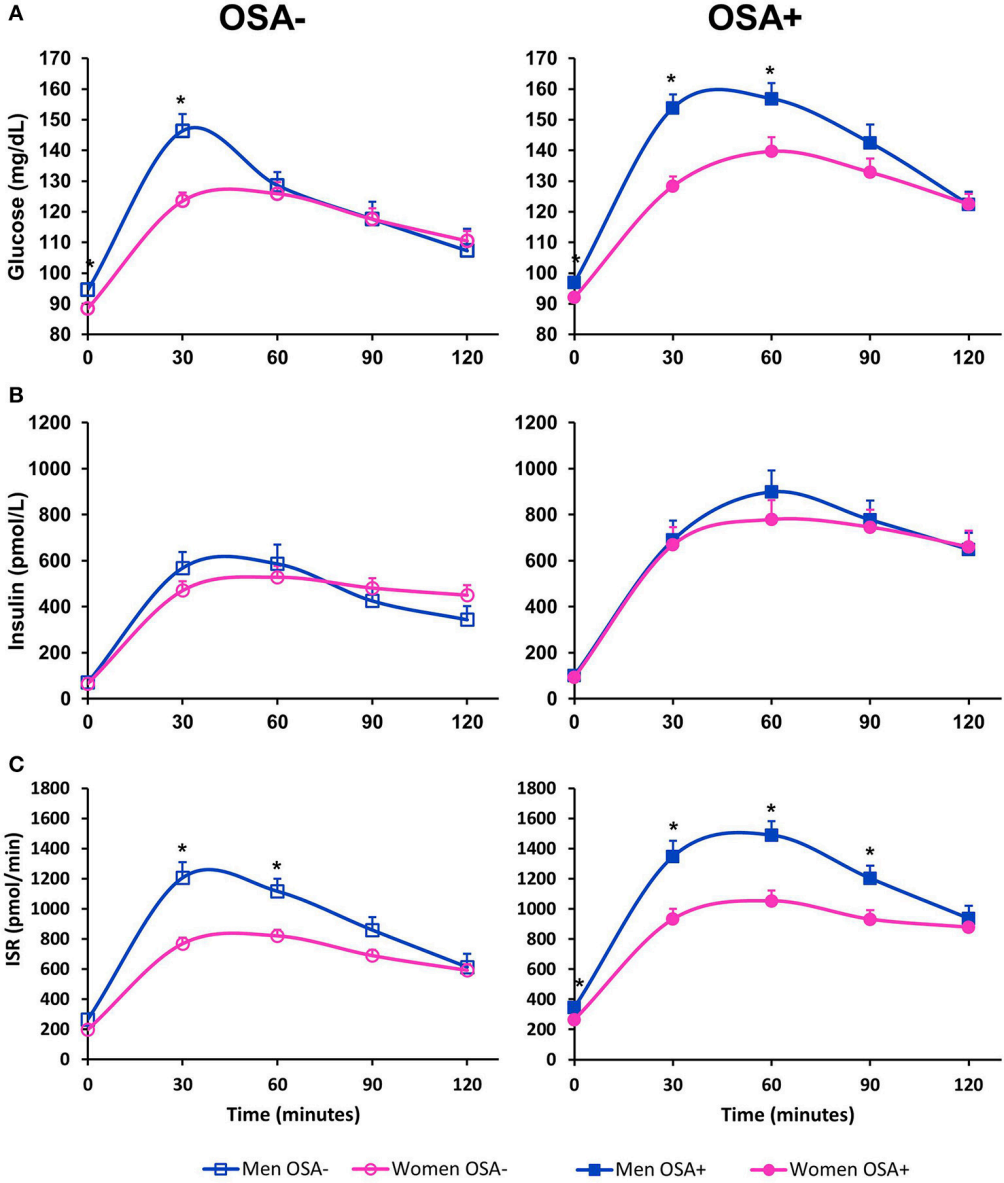
Oral glucose tolerance test in men and women with and without OSA. **(A)** Illustrates plasma glucose, **(B)** illustrates serum insulin and **(C)** illustrates insulin secretion rate (ISR) as measured during a 2-h 75-g oral glucose challenge. Data are represented as mean + SEM. ^*^denotes an unadjusted *p*-value < 0.05 from a student's *t*-test for variables with normal distribution, and Wilcoxon test for variables that are not normally distributed.

Figure 1 illustrates profiles of plasma glucose, serum insulin, and ISR from the OGTT. In unadjusted analyses, men with OSA had a significantly higher fasting, 30-min and 1-h glucose levels compared to women with OSA. Men with OSA had significantly higher AUC_Glucose_ than women with OSA (*p* = 0.005). Although men without OSA had a higher fasting and 30-min glucose levels compared to women without OSA, the AUC_Glucose_ was similar between men and women without OSA (*p* = 0.204). In unadjusted analysis, the AUC_ISR_ was significantly greater in men with OSA compared to women with OSA (*p* = 0.0069), as well as in men without OSA compared to women without OSA (*p* = 0.0030).

Tables 3, 4 summarize metabolic differences between men and women. In subjects without OSA, men had a higher fasting glucose in both unadjusted and adjusted comparisons. There were no significant differences between men and women without OSA in any of the measures of beta-cell function, measures of insulin sensitivity, and glucose tolerance. In contrast, in subjects with OSA, men had significantly higher fasting glucose, 1-hr glucose, and AUC_Glucose_. Men with OSA secreted more insulin compared to women with OSA, as evidenced by a significantly higher AUC_ISR_. These differences remained significant after adjusting for covariates. Men with OSA had significantly lower beta-cell function as assessed by the DI_Oral_ even after adjusting for covariates.

**Table 3:** Fasting metabolic measures.

**Variable**	**Women OSA-** **(*n* = 51)**	**Men OSA-** **(*n* = 15)**	**Unadjusted *p*** **(*n* = 66)**	Adjusted p^*^ **(*n* = 66)**	**Women OSA+** **(*n* = 39)**	**Men OSA+** **(*n* = 40)**	**Unadjusted *p*** **(*n* = 79)**	Adjusted p^*^ **(*n* = 79)**
Fasting glucose (mg/dL)	88.5 ± 0.9	94.5 ± 2.3	**0.0039**	**0.0028**	92.1 ± 1.0	97.0 ± 1.0	**0.0007**	**0.0010**
Fasting insulin (pmol/L)	49 (31–90)	52 (21–97)	0.963	0.952	83 (56–118)	89 (48–149)	0.814	0.416
Fasting ISR^a^ (pmol/min/m^2^)	192 (144–240)	228 (169–340)	0.102	0.224	212 (166–334)	292 (207–453)	**0.0354**	0.115
HOMA–IR (mIU/mmol)	1.61 (0.98–2.89)	1.78 (0.64–3.34)	0.842	0.736	2.77 (1.92–3.70)	2.98 (1.54–5.21)	0.580	**0.255**
HOMA–%B (mIU/mmol)	120 (71–190)	82 (48–151)	0.335	0.318	161 (88–246)	126 (85–215)	0.430	0.800
HbA1c (%)	5.44 ± 0.04	5.43 ± 0.07	0.875	0.826	5.63 ± 0.04	5.59 ± 0.06	0.619	0.582

Data are given as mean ± SEM or median (interquartile range) for variables that are not normally distributed.

*Unadjusted p-values are from a student's t-test for variables with normal distribution, and Wilcoxon test for variables that are not normally distributed*.

* *p-values are from a multiple regression model adjusting for age, BMI, race, and natural log (Ln)AHI. P-values in bold are statistically significant*.

a *Multiple regression models for ISR did not adjust for BMI because the calculation for ISR includes an adjustment for body surface area*. *ISR, Insulin Secretion Rate; HOMA-IR, Homeostatic Model Assessment Insulin Resistance; HOMA-%B, Homeostatic Model Assessment Beta Cell Function*.

**Table 4:** Metabolic measures derived from response to oral glucose.

**Variable**	**Women OSA-** **(*n* = 51)**	**Men OSA-** **(*n* = 15)**	**Unadjusted *p*** **(*n* = 66)**	Adjusted p^*^ **(*n* = 66)**	**Women OSA+** **(*n* = 39)**	**Men OSA+** **(*n* = 40)**	**Unadjusted *p*** **(*n* = 79)**	Adjusted p^*^ **(*n* = 79)**
2-h Glucose (mg/dL)	110.5 ± 3.3	107.3 ± 7.2	0.660	0.904	122.5 ± 3.3	122.4 ± 4.2	0.978	0.512
1-h Glucose (mg/dL)	125.9 ± 3.7	128.6 ± 4.3	0.709	0.718	139.7 ± 4.6	156.9 ± 5.0	**0.0135**	**0.0225**
AUC_Glucose_	13,995 ± 312	14,805 ± 472	0.204	0.212	15,247 ± 375	16,893 ± 427	**0.0050**	**0.0066**
AUC_Insulin_	42,920 (28,179–66,307)	54,429 (35,477–70,750)	0.520	0.786	56,8312 (35,367–119,603)	70,813 (42,415–106,936)	0.691	0.539
${\text{AUC}}_{\text{ISR}}^{\text{a}}$	2,867 (2,213–3,630)	4,094 (3,384–5,111)	**0.0030**	0.055	3,993 (2,833–5,197)	4,973 (4,087–6,409)	**0.0069**	**0.0319**
AUC_Insulin/Glucose_	57.9 (40.4–85.8)	68.4 (42.1–76.7)	0.807	0.918	71.6 (46.1–129.6)	77.0 (50.7–117.8)	0.841	0.970
DI_Oral_	3.60 (2.20–6.74)	2.94 (2.11–7.67)	0.481	0.529	3.15 (2.24–4.72)	2.16 (1.09–3.27)	**0.0090**	**0.0116**
IGI	187 (122–290)	179 (132–205)	0.391	0.493	258 (127–425)	149 (81–299)	**0.0314**	0.123
Matsuda index	5.10 (3.21–8.08)	4.17 (2.61–7.45)	0.592	0.643	3.21 (1.86–4.54)	2.44 (1.58–5.16)	0.424	0.226

Data are given as mean ± SEM or median (interquartile range) for variables that are not normally distributed.

Unadjusted p values are from a student's t-test for variables with normal distribution, and Wilcoxon test for variables that are not normally distributed.

* *p-values are from a multiple regression model adjusting for age, BMI, race, and LnAHI. P-values in bold are statistically significant*.

a *Multiple regression models for AUC_ISR_ did not adjust for BMI because the calculation for ISR includes an adjustment for body surface area*.

*AUC, Area under the curve; ISR, Insulin Secretion Rate; DI_Oral_, Oral disposition index; IGI, Insulinogenic index*.

## Discussion

Our study rigorously examines sex-specific differences in the impact of OSA on glucose metabolism in relatively young overweight and obese subjects without diabetes. There were no significant differences in glucose tolerance and beta-cell function between men and women without OSA. In contrast, men with OSA had worse glucose tolerance than women with OSA despite secreting more insulin. As such, the adverse impact of OSA on beta-cell responsiveness is of greater magnitude in men, which may result in an overall greater risk of developing type 2 diabetes. Higher glucose levels and insulin responses during the OGTT (i.e., 1-h glucose and the shape of the plasma glucose curve) are indeed related to the risk of developing type 2 diabetes, particularly if the 2-h plasma glucose does not return to or below the fasting plasma glucose levels (23–25). A slower rate of decrease in plasma glucose concentration during an OGTT is indicative of increased insulin resistance and/or impaired beta-cell responsiveness, both of these being important risk factors for future type 2 diabetes.

The underlying pathogenesis of impaired glucose metabolism due to OSA is not fully understood but factors such as activation of the sympathetic nervous system, intermittent hypoxemia, oxidative stress, and low-grade systemic inflammation have been implicated. In a study of 118 nondiabetics recruited from the community who underwent a frequently sampled intravenous glucose tolerance test, the severity of OSA as measured by the AHI was independently associated with insulin sensitivity after controlling for age, sex, race and percent body fat. Moreover, the acute insulin response to glucose, a measure of beta-cell function, did not increase across OSA severity categories. Together these findings suggest that the increased diabetes risk in OSA is associated with increased insulin resistance without adequate compensation by the beta-cell (26). The sex-specific differences of the impact of OSA on glucose metabolism, however, remains mostly unexplored as most studies have predominantly included men or only women or did not take sex differences into account (4–10, 27, 28). A few cross-sectional epidemiologic studies have suggested that markers of OSA, namely observed apneas and habitual snoring, are independently associated with type 2 diabetes or prediabetes in women only (29–32). These studies were questionnaire based and lacked objective polysomnographic evaluation. In contrast, we quantified the presence and severity of OSA using the gold standard in-laboratory polysomnography. Our findings of increased risk of type 2 diabetes with OSA in men are consistent with findings from the largest nationwide health claims database analysis which revealed a higher prevalence of type 2 diabetes in men with OSA compared to women with OSA (33). In a Swedish clinic-based longitudinal study with 16 years of follow up, OSA was independently associated with incident type 2 diabetes in women only (34). However, this study was limited due to lack of full in-lab polysomnography, it included only 10 women with OSA and the presence of incident type 2 diabetes was obtained by questionnaires mailed to the patients. The questionnaire based nature of the study and lack of OGTT precludes any inferences regarding sex-specific mechanistic differences by which OSA may impact glucose metabolism.

A number of population-based and clinic-based studies have reported a stronger association between OSA and hypertension in men than in women (35–37). There is a paucity of studies examining sex differences of the adverse metabolic impact of OSA. Our cross-sectional analysis suggests that the adverse impact of OSA on beta-cell function is more prominent in men. Harsch and colleagues performed hyperinsulinemic euglycemic clamps after 2 days of all-night CPAP therapy in the sleep laboratory in 40 nondiabetic subjects (36 men and 4 women) with severe OSA. Although they did not measure beta-cell function, they demonstrated significant improvement in insulin sensitivity after only 2 nights of effective all-night CPAP therapy (38). Clinical trials assessing the impact of CPAP therapy on glucose metabolism in patients with OSA and type 2 diabetes, on the other hand, have yielded mixed results, in part due limited adherence to CPAP therapy (11). However, clinical trials with higher CPAP adherence in patients with either type 2 diabetes (39, 40) or prediabetes (41, 42) have found that therapeutic CPAP improves glycemic control or insulin sensitivity compared to the control group. These findings suggest that in order to derive metabolic benefits from CPAP therapy, treatment should include the great majority of the sleep period (43).

Our study has several limitations. First and foremost, its cross-sectional design does not establish the direction of causality. Second, we used derived measures of insulin resistance, glucose tolerance and beta-cell function from an OGTT alone. Although some of the derived measures of beta-cell function have been previously validated (18, 44), there is no clear consensus on how best to measure beta-cell function based on an OGTT. We therefore explored several derived measures of beta-cell function. Undoubtedly, additional studies with more detailed and intensive assessments of beta-cell function and insulin sensitivity are needed to better elucidate mechanistic pathways. Third, our study includes one night of sleep in the laboratory and therefore does not take into account variability in sleep patterns as well as participants' habitual sleep duration which can influence glucose metabolism. Fourth, although we excluded post-menopausal women, metabolic testing was not standardized to a particular phase of the menstrual cycle. Lastly, we did not assess measures of body fat distribution. This may be relevant given the established sex differences in fat distribution.

In summary, the adverse impact of OSA on beta-cell responsiveness is greater in men, which may result in an overall higher risk of future development of type 2 diabetes compared to women.

## Author contributions

BM and EV designed the protocol. LM, KT, and RL recruited subjects and collected data. KT, DE, EV, and BM analyzed the data. BM drafted the manuscript. KT, EV, DE, and BM reviewed and edited the manuscript.

### Conflict of interest statement

BM is supported by National Institutes of Health grant R01HL119161 and is supported by the Merck Investigator Studies Program. EV is the principal investigator of an investigator-initiated study sponsored by Philips/Respironics. The remaining authors declare that the research was conducted in the absence of any commercial or financial relationships that could be construed as a potential conflict of interest.
